# Prevalence of Soil-Transmitted Helminth and *Schistosoma mansoni* Infection and Their Associated Factors among Hiruy Abaregawi Primary School Children, Rural Debre Tabor, North West Ethiopia: A Cross-Sectional Study

**DOI:** 10.1155/2020/2521750

**Published:** 2020-01-29

**Authors:** Lemma Workineh, Teklehaimanot Kiros, Shewaneh Damtie, Tesfaye Andualem, Bizualem Dessie

**Affiliations:** Debre Tabor University, College of Medicine and Health Sciences, Department of Medical Laboratory Science, Debre Tabor, Ethiopia

## Abstract

**Background:**

In Ethiopia, 25.3 and 12.3 million school-age children are living in soil-transmitted helminth and schistosomiasis endemic areas, respectively. The school children are at risk for both soil-transmitted helminths and *Schistosoma mansoni* due to juvenile activities like walking barefoot, playing with dirty objects that might be contaminated with feces, and fetching of unclean water for drinking. There are no data that indicate the status of soil-transmitted helminths and *Schistosoma mansoni* among children at Hiruy Abaregawi primary school. Therefore, the main objective of this study was to determine the prevalence of soil-transmitted helminth and *Schistosoma mansoni* infection among Hiruy Abaregawi primary school children.

**Methods:**

A cross-sectional study was conducted from March to April, 2019, at Hiruy Abaregawi primary school, Rural Debre Tabor, North West Ethiopia. A total of 340 students were included in the study. Informed written consent was obtained from the children's parent. Systematic sampling technique was used to select the children. About 2 grams of stool samples was collected and transported to Debre Tabor University Microbiology and Parasitology Teaching Laboratory to conduct the Kato-Katz technique. Data were analyzed using SPSS version 23. Variables with a *p* value < 0.05 were considered statistically significant.

**Results:**

The prevalence of soil-transmitted helminths and *Schistosoma mansoni* was 51/340 (15%). Among the identified parasites, *Ascaris lumbricoides* accounts for 28 (8.2%), hookworm 13 (3.8%), *Trichuris trichiura* 4 (1.2%), and *Schistosoma mansoni* 6 (1.8%). In this study, 24 (7%) of *Ascaris lumbricoides*-, 11 (3.2%) of hookworm-, 4 (1.2%) of *Trichuris trichiura*-, and 5 (1.5%) of *Schistosoma mansoni*-infected children showed light infections and no heavy infection in both soil-transmitted helminths and *Schistosoma mansoni* was observed. Finger nail trimming status, hand washing before eating, availability of toilet at home, educational level of students, and sex of students were factors associated with soil-transmitted helminth infection. *Conclusion and Recommendations*. In this study, the low prevalence of soil-transmitted helminths and *Schistosoma mansoni* was observed. The combination of regular mass deworming program and health information on risk factors should be strengthened for the prevention and control of soil-transmitted helminth infection.

## 1. Introduction

Both soil-transmitted helminth (STH) infection and schistosomiasis are considered two of the major public health important neglected tropical diseases (NTDs) and cause chronic malnutrition, stunted growth, cognitive impairment, and anemia [1, 2]. STH infection is caused by *Ascaris lumbricoides* (*A. lumbricoides*), *Trichuris trichiura* (*T. trichiura*), and hookworm whereas schistosomiasis is caused by the medically important *Schistosoma* species (*Schistosoma* spp.) such as *Schistosoma mansoni* (*S. mansoni*), *Schistosoma haematobium* (*S. haematobium*), and *Schistosoma japonicum* (*S. japonicum*) [3]. Infection with STHs occurs through accidental ingestion of *A*. *lumbricoides* and *T. trichiura* eggs and larval penetration of the skin by hookworm present in contaminated soil while schistosomiasis is transmitted by cercarial skin penetration from fecally contaminated water [4].

STH infection and schistosomiasis infect more than 1.5 billion people and 240 million people, respectively, [5]. Globally, over 267 million preschool-age children and 568 million school-age children live in areas where STHs are intensively transmitted and are in need of treatment and preventive interventions [6]. In sub-Saharan Africa, about 198, 192, 173, and 162 million people are infected with hookworm, *Schistosoma* spp., *A. lumbricoides*, and *T. trichiura*, respectively, [7, 8].

In Ethiopia, the number of people living in STH endemic areas is estimated to be 81 million, comprising of 9.1 million preschool-age children, 25.3 million school-age children, and 44.6 million adults whereas about 38.3 million people are living in schistosomiasis endemic areas, comprising of 34.4 million preschool children, 12.3 million school-age children, and 21.6 million adults [9].

In the rural parts of Debre Tabor town, most of the school children are at risk for both STH and *S. mansoni* infection due to exposure to risky juvenile activities like walking on bare foot, playing with dirty objects that could be contaminated with feces, and drinking of unclean water. There are no data that indicate the prevalence of STH and *S. mansoni* infection among the school children in the rural parts of Debre Tabor town. So this study will give clue for the stakeholders and policy makers on how to control and prevent STH and *S. mansoni* infection among the school children in the rural parts of the Debre Tabor town. Therefore, the main objective of this study was to determine the prevalence of STHs and *S. mansoni* among Hiruy Abaregawi primary school children.

## 2. Methods and Materials

### 2.1. Study Area and Period

A cross-sectional study was conducted from March to April, 2019, on STH and *S. mansoni* infections among Hiruy Abaregawi primary school children, rural Debre Tabor, North West Ethiopia. Hiruy Abaregawi primary school is located at the eastern part of rural Debre Tabor town. Debre Tabor is the capital town of the South Gonder zone. It is 102 km far from Bahir Dar, which is the capital city of Amhara regional sate and 666 km from Addis Ababa. The total numbers of students learning at the school were 1325. Out of this, 650 and 675 were females and males, respectively.

### 2.2. Sample Size and Sampling Technique

There are 8 primary schools in Debre Tabor town. Four of them are found in the center of the town and excluded because it is assumed that the prevalence rate decreases due to good sanitation and personal hygiene in the town. Hiruy Abaregawi primary school was randomly selected from the 4 rural Debre Tabor primary schools. Sample size was calculated by using 27.9% previous prevalence [10]. By using the single proportion formula, 95% CI, and 5% margin of error with a 10% nonresponse rate, 340 students were included in the study. The total number of children from grades 1-8 was 1325. There were 8 classes and 4 sections in each class in the school. Two sections were selected from each class by using a systematic sampling technique. Then, the number of children sampled from each section was proportionally allocated by dividing the number of students in the selected section to the total number of students in the school, and finally, the proportion was multiplied by the total sample size. Then, after determining the number of students from the selected section, a systematic sampling technique was used to select children.

### 2.3. Eligibility Criteria

The students who were willing to participate, provided sufficient stool samples, and gave informed assent and consent from their parents were included while the students who had taken helminth chemotherapy before data collection were excluded.

### 2.4. Parasitological Stool Sample Collection, Processing, and Examination

The school children were given a plastic stool cup and informed to provide about 2 grams of stool sample. Then, the stool samples were preserved with 10% formalin and packed in a plastic bag to transport to Debre Tabor University Microbiology and Parasitology Teaching Laboratory to conduct the Kato-Katz technique. Two Kato slides per stool sample were prepared using a fixed quantity of sieved 41.7 mg of stool on a punched template [11]. Each stool sample was placed on a newspaper and covered with a nylon screen. The stool was forced through the screen using a plastic spatula, and a portion of the sieved material was filled in the respective template that had been placed on the weighed labeled microscope slides. With a spatula, the surface was leveled and excess stool from the edge of the template hole was carefully removed. Then after, the templates were carefully removed from the slides by lifting it vertically. Finally, the slides were observed within one hour under the microscope at a magnification of ×10 objective.

The total numbers of eggs were expressed as eggs per gram (EPG) of stool. EPG was calculated to classify the infection intensity as light, moderate, and heavy infection [12]. The intensity of STH and *S. mansoni* infection is defined as light, moderate, and heavy infections, respectively, as follows: *A. lumbricoides*: 1 EPG to 4999 EPG, 5000 EPG to 49999 EPG, and ≥50000 EPG; *T. trichiura*: 1 EPG to 999 EPG, 1000 EPG to 9999 EPG, and ≥10000 EPG; hookworm: 1 to 1999 EPG, 2000 EPG to 3999 EPG, and ≥4000 EPG; *S. mansoni*: 1 EPG to 99 EPG, 100 EPG to 399 EPG, and ≥400 EPG.

Nine laboratory personnel (4 persons for Kato-Katz preparation and 5 persons for microscopic examinations) were involved in the laboratory. Moreover, data on sociodemographic characteristics of the study participants and associated factors of STH and S*. mansoni* infection were collected by trained health professionals with standardized pretested questionnaires.

### 2.5. Quality Assurance and Quality Control

In the preanalytical phase, students were oriented on how to collect stool samples; 1 gram of stool was mixed with 3 ml of formalin preservative; sample packaging and transportation, soaking of cellophane in glycerol-malachite green solution for 24 hours, and Kato-Katz preparation such as filling stool in the hole of the templates by avoiding overfill and under fill were assured. In the analytical phase, the prepared slides were systematically examined by zigzag form under 10x and 40x microscope objectives. In the postanalytical phase, calculation of egg per gram of stool and documentation were assured.

The performance of Kato-Katz and reagents was checked by positive and negative samples. Finally, ten percent of the examined Kato-Katz slides were randomly selected and reexamined at the end by a qualified laboratory technologist from Amhara Public Health Institute who was blind for the first result. Then, after checking 10% of the slides, no discordant result was observed.

### 2.6. Data Management and Analysis

Data were analyzed using SPSS version 23. Descriptive statistics was done to calculate sociodemographic characteristics and the prevalence rate. The associated factors of STH infection were analyzed by binary logistic regression. Firstly, bivariate logistic regression analysis was done, and then to control the possible confounding factors, variables with a *p* value < 0.2 were adjusted by multivariate logistic by stepwise variable selection. Finally, variables with a *p* value < 0.05 were considered statistically significant.

### 2.7. Operational Definition

The different prevalence levels are categorized as follows:

*Low prevalence:* prevalence of less than 50% for STHs and less than or equal to 10% for schistosomiasis [1]

*Moderate prevalence:* prevalence of greater than or equal to 50% but less than 70% for STHs and greater than or equal to 10% but less than 50% for schistosomiasis

*High prevalence:* prevalence of greater than or equal to 70% for STH and greater than or equal to 50% for schistosomiasis

### 2.8. Ethical Considerations

Ethical clearance was obtained from Debre Tabor University, College of Medicine and Health Sciences, and permission letter to conduct the study was obtained from the Debre Town education department and Hiruy Abaregawi primary school director office. Additionally, after explaining the importance of the study briefly, an informed written consent was obtained from parents of the school children. Finally, those students who were positive for STHs and *S. mansoni* were linked to Debre Tabor town health department for treatment by appropriate antihelminthic drugs.

## 3. Results

### 3.1. Sociodemographic Characteristics of Study Subjects

A total of 340 school children were included in this study. Out of these, 194 (50.6%) and 146 (49.4%) were males and females, respectively. The minimum age of the study participants was 5 years, and the maximum age was 18 years with a mean age of 10.6 years. The majority of the study participants, 189 (55.6%), were in the age group of 10-14 years. The majority of the study subjects comprising of 241 (77%) were from a rural area (Table 1), and the remaining were from an urban area.

### 3.2. The Prevalence of STH and *S. mansoni* Infection

Out of 340 school children, 51 students were infected with STHs and *S. mansoni* with an overall prevalence of 15% (CI: 11.2%-18.8%). The prevalence of STHs and *S. mansoni* among males was 16% while 13% of females were infected by STHs and *S. mansoni*. The high prevalence of STHs and *S. mansoni* was observed in the age group of 10-14 years with the prevalence of 16%, and the least prevalence was observed in the age group of 15-18 years with 11.7%. About 20% of students infected by STHs and *S. mansoni* were from grades 1-4 whereas the least 10.6% infected children were from grades 5-8. The students infected by STHs and S. *mansoni* who were from the rural area were 15.3% as compared to those who were from the urban area (Table 1).

**Figure 1:**
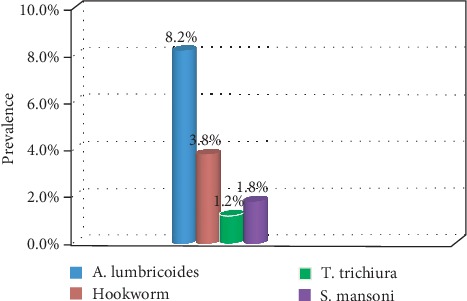
Distributions of STH and *S. mansoni* infections among Hiruy Abaregawi primary school children from March to April, 2019, Debre Tabor town, North West Ethiopia.

Among the identified parasites, A*. lumbricoides* accounted for 28 (8.2%), hookworm 13 (3.8%), *T. trichiura* 4 (1.2%), and *S. mansoni* 6 (1.8%) (Figure 1).

### 3.3. Infection Intensity of STHs and *S. mansoni*

In this study, 24 (7%) of *A. lumbricoides*-, 11 (3.2%) of hookworm-, 4 (1.2%) of *T. trichiura*-, and 5 (1.5%) of *S. mansoni*-infected children showed light infections (Table 2).

After multiplying the number of eggs per slides by 24 as a factor, the mean intensity of *A. lumbricoides*, hookworm, *T. trichiura*, and *S. mansoni* was 1148.6 EPG, 557.5 EPG, 54 EPG, and 68 EPG, respectively.

### 3.4. Analysis of Factors Associated with STH Infection

All variables were first analyzed by bivariate logistic regression. In bivariate analysis, the age of the respondent, shoe-wearing habit, hand washing after the toilet, and water source were not associated with STH infection (*p* > 0.05). However, the educational level of the students, residence, trimming of finger nail status, hand washing before eating a meal, and latrine availability were factors associated with STH infection (*p* < 0.05) (Table 3).

All bivariate results that had a *p* value < 0.2 were subjected to a multivariate logistic regression model to control possible confounding factors. After adjustment, the shoe-wearing habit was not significant with STH (*p* > 0.05). Female students were 0.41 less likely to be infected by STHs than male students (AOR = 0.41, CI = 0.010‐0.172). The odds of being infected by STHs among students from the rural area were 6.7 as compared to those from the urban areas (AOR = 6.733, CI = 2.119‐21.394). The children who did not trim their finger nail were 5 times more likely to be infected by STH than those who did. Those students who did not wash their hands before eating a meal were 5.4 more likely to be infected than who washed their hands before eating a meal always (AOR = 5.496, CI = 2.012‐15.012), and the students who washed sometimes were 4.5 more likely to be infected than those who washed always (AOR = 4.506, CI = 1.321‐15.370). The odds of being infected by STHs in those children who had no latrine at their home were 3.948 as compared to those who had toilet availability (AOR = 3.948, CI = 1.510‐10.325) (Table 4).

## 4. Discussions

The finding of this study showed that the overall prevalence STHs and *S. mansoni* was 15% (CI: 11.2%-18.8%). The prevalence of STHs was 13.2% giving 8.2% (CI = 5.3%‐11.2%) of *A. lumbricoides*, 3.8% (CI = 2.1%‐6.2%) of hookworm, and 1.2% (CI = 0.3%‐2.4%) of *T. trichiura*. The prevalence of *A. lumbricoides* and *T. trichiura* in this study was similar to the study conducted in Libo kem district [10] with a prevalence of 11% and 1.6%, respectively.

Moreover, this study was different from other previous studies such as 39.8% of *A. lumbricoides* and 6.1% of *T. trichiura* among primary school children, in North Gonder [13]; 0.2%, 14.75%, and 0.2% of *A. lumbricoides*, hookworm, and *T. trichiura*, respectively, in Dera district [14]; 12.7% of *A. lumbricoides*, 43.4% of hookworm, and 10% of *T. trichiura*, in Zegie peninsula [15]; 31.8% of hookworm, 29.4% of *A. lumbricoides*, and 3.1% of *T. trichiura*, in Lake Tana [16]; 37.1% of hookworm, 16.1% *A. lumbricoides*, and 0.8% of *T. trichiura*, in Sanja town [17]; 2.6% of *A. lumbricoides*, 2.85% of hookworm, 2.6% of *T. trichiura*, in Tach Armachiho district [18]; and 33.3% of hookworm, 15.5% of *A. lumbricoides*, and 1.6% of *T. trichiura*, in Motta town [19].

The variations in the prevalence of STHs between the current study and previous study might be due to the difference in sanitation status, temperature difference, and variation in socioeconomic factors. In this study, the lower prevalence in STHs could be due to 258 (75.8%) of students who had latrine availability at their home, 305 (89.7%) of students who trimmed their finger nail, and 223 (65.5%) of the students who wore shoes always (Table 3). The average temperature in Debre Tabor is 14.8°C which could make a low prevalence of STH infection in this study. It is well documented that a warmer temperature facilitates infectivity and development of parasite [20].

In this study, the prevalence of *S. mansoni* was 1.8% (CI = 0.6%‐3.2%). This finding is consistent with 2.4% in Dera district [14]. However, it is lower than 14.3% in Lake Tana [16], 89.9% in Sanja town [17], 56.6% in Tach Armachiho district [18], and 29.9% in Zegie peninsula [15]. The difference could be due to low risk factors like absence of a water body for swimming and irrigational activities in the study area. Moreover, the high-altitude nature of Debre Tabor town could decrease the prevalence of *S. mansoni* in the study area because it is well documented that high altitude decreases the distribution of schistosomiasis [21].

With regard to infection intensity, no heavy infection in both STHs and *S. mansoni* was observed. About 7% of *A. lumbricoides*, 3.2% of hookworm, 1.2% of *T. trichiura*, and 1.5% of *S. mansoni* showed light infections whereas 1.2% of *A. lumbricoides*, 0.6% of hookworm, and 0.3% of *S. mansoni* showed moderate infections. This finding was different from the study conducted in Tach Armachiho district [18] and in Sanja town [17]. This could be due to the difference in the exposure rate and immunity to reinfection.

In this study, STH infection was associated with finger nail trimming status, hand washing before eating a meal, availability of toilet at home, the educational level of students, and sex of students. These findings were in line with the study done in Tach Armachiho district [18] and Motta town [19].

## 5. Conclusion and Recommendations

In this study, the low prevalence of STHs and *S. mansoni* was observed. The combination of regular mass deworming program and health information on risk factors should be strengthened for the prevention and control of STH infection.

## Figures and Tables

**Table 1:** STH and *S. mansoni* infections with sociodemographic factors among Hiruy Abaregawi primary school children from March to April, 2019, Debre Tabor Town, North West Ethiopia.

Sociodemographic characteristics	STH and *S. mansoni* infection		
	Positive	Negative	Total
Sex			
Male	32 (16.0%)	162 (84%)	194 (57.1%)
Female	19 (13.0%)	127 (87%)	146 (42.9%)
Age			
5-9	17 (14.5%)	100 (85.5%)	117 (34.4%)
10-14	30 (16.0%)	159 (84.0%)	189 (55.6%)
15-18	4 (11.7%)	30 (88.3%)	34 (10.0%)
Education level of the students			
Grades 1-4	32 (20.0%)	129 (80.0%)	161 (47.4%)
Grades 5-8	19 (10.6%)	160 (89.4%)	179 (52.6%)
Residence			
Rural	37 (15.3%)	204 (84.7%)	241 (70.9%)
Urban	14 (14.0%)	85 (86.0%)	99 (29.1%)
Father's educational status			
Illiterate	29 (17.0%)	140 (83.0%)	169 (49.7%)
1-8	17 (13.0%)	115 (87.0%)	132 (38.8%)
High school and above	5 (12.8%)	34 (87.2%)	39 (11.5%)
Mother's educational status			
Illiterate	39 (17.6%)	182 (82.4%)	22 (65.0%)
1-8	10 (8.8%)	103 (91.2%)	113 (33.3%)
High school and above	1 (6.2%)	15 (93.8%)	16 (4.7%)

**Table 2:** Intensity of STH and *S. mansoni* infections among Hiruy Abaregawi primary school children from March to April, 2019, Debre Tabor town, North West Ethiopia.

Infection intensity	*A. lumbricoides* No. (%)	Hookworm No. (%)	*T. trichiura* No. (%)	*S. mansoni* No. (%)
Light infection	24 (7.0)	11 (3.2)	4 (1.2)	5 (1.5)
Moderate infection	4 (1.2)	2 (0.6)	0 (0.0)	1 (0.3)
Heavy infection	0 (0.0)	0 (0.0)	0 (0.0)	0 (0)
Total positive	28 (8.2)	13 (3.8)	4 (1.2)	6 (1.8)
Negative	312 (91.8)	327 (96.2)	336 (98.8)	334 (98.2)

Total	340 (100)	340 (100)	340 (100)	340 (100)

**Table 3:** Bivariate analysis of association factors of STH infection among Hiruy Abaregawi primary school children from March to April, 2019, Debre Tabor town, North West Ethiopia.

Associate factors of STHs	STH infections		COR (95% CI)	*p* value
	Positive	Negative		
Sex				
Male	28	144	1	
Female	17	151	0.579 (0.304-1.103)	0.097
Age				
5-9	15	102	1.520 (0.413-5.593)	0.529
10-14	27	162	1.722 (0.492-6.030)	0.395
15-18	3	31	1	
Educational level of the students				
Grades 1-4	28	133	2.100 (1.003-3.853)	0.034
Grades 5-8	17	162	1	
Residence				
Rural	35	206	1.512 (0.718-3.187)	0.190
Urban	10	89	1	
Water source				
Tap water	7	59	1	
Spring	9	80	0.948 (0.334-2.692)	0.920
Well water	29	156	1.567 (0.651-3.770)	0.316
Trimming of finger nail				
Yes	35	270	1	
No	10	25	3.060 (1.368-6.961)	0.007
Hand washing before eating a meal				
No	12	36	3.239 (1.409-7.447)	0.006
Sometimes	18	88	2.264 (1.088-4.708)	0.029
Always	15	171	1	
Hand washing after using the toilet				
No	16	49	1.299 (0.619-2.716)	0.490
Sometimes	12	95	0.719 (0.328-1.578)	0.411
Always	17	161	1	
Latrine availability				
Yes	28	230	1	
No	17	65	2.14 (1.108-4.167)	0.024
Shoe-wearing habit				
No	5	13	2.934 (0.970-8.875)	0.057
Sometimes	13	76	1.305 (0.0640-2.660)	0.464
Always	17	206	1	

**Table 4:** Multivariate analysis of association factors of STH infection among Hiruy Abaregawi primary school children from March to April, 2019, Debre Tabor town, North West Ethiopia.

Associate factors of STHs	STH infections		COR (95% CI)	*p* value
	Positive	Negative		
Sex				
Male	28	144	1	
Female	17	151	0.410 (0.010-1.172)	0.000
Educational level of the students				
Grades 1-4	28	133	3.281 (1.136-9.479)	0.028
Grades 5-8	17	162	1	
Residence				
Rural	35	206	6.733 (02.119-9.479)	0.001
Urban	10	89	1	
Trimming of finger nail				
Yes	35	270	1	
No	10	25	5.154 (1.300-20.437)	0.020
Hand washing before eating a meal				
No	12	36	5.496 (2.012-15.012)	0.001
Sometimes	18	88	4.506 (1.321-15.370)	0.016
Always	15	171	1	
Latrine availability				
Yes	28	230	1	
No	17	65	3.948 (1.510-10.325)	0.005
Shoe-wearing habit				
No	5	13	3.483 (0.836-14.507)	0.086
Sometimes	13	76	1.521 (0.547-4.230)	0.422
Always	17	206	1	

## Data Availability

The data used to support the findings of this study are included within the article.

## Additional Points

*Limitation.* We were unable to do an analysis of associated factors with *S. mansoni* due to the low prevalence of *S. mansoni* in the study area.
